# Prediction of Lung Shunt Fraction for Yttrium-90 Treatment of Hepatic Tumors Using Dynamic Contrast Enhanced MRI with Quantitative Perfusion Processing

**DOI:** 10.3390/tomography8060224

**Published:** 2022-11-03

**Authors:** Qihao Zhang, Kyungmouk Steve Lee, Adam D. Talenfeld, Pascal Spincemaille, Martin R. Prince, Yi Wang

**Affiliations:** 1 Department of Biomedical Engineering, Cornell University, Ithaca, NY 14853, USA; 2 Radiology, Weill Cornell Medicine, New York, NY 10065, USA

**Keywords:** dynamic contrast enhanced MRI, lung shunting fraction, Yttrium-90 treatment, hepatocellular carcinoma, quantitative transport mapping

## Abstract

There is no noninvasive method to estimate lung shunting fraction (LSF) in patients with liver tumors undergoing Yttrium-90 (Y90) therapy. We propose to predict LSF from noninvasive dynamic contrast enhanced (DCE) MRI using perfusion quantification. Two perfusion quantification methods were used to process DCE MRI in 25 liver tumor patients: Kety’s tracer kinetic modeling with a delay-fitted global arterial input function (AIF) and quantitative transport mapping (QTM) based on the inversion of transport equation using spatial deconvolution without AIF. LSF was measured on SPECT following Tc-99m macroaggregated albumin (MAA) administration via hepatic arterial catheter. The patient cohort was partitioned into a low-risk group (LSF ≤ 10%) and a high-risk group (LSF > 10%). Results: In this patient cohort, LSF was positively correlated with QTM velocity |u| (r = 0.61, F = 14.0363, *p* = 0.0021), and no significant correlation was observed with Kety’s parameters, tumor volume, patient age and gender. Between the low LSF and high LSF groups, there was a significant difference for QTM |u| (0.0760 ± 0.0440 vs. 0.1822 ± 0.1225 mm/s, *p* = 0.0011), and Kety’s $K^{trans}$ (0.0401 ± 0.0360 vs 0.1198 ± 0.3048, *p* = 0.0471) and $V_{e}\;$(0.0900 ± 0.0307 vs. 0.1495 ± 0.0485, *p* = 0.0114). The area under the curve (AUC) for distinguishing between low LSF and high LSF was 0.87 for |u|, 0.80 for $V_{e}$ and 0.74 for $K^{trans}$. Noninvasive prediction of LSF is feasible from DCE MRI with QTM velocity postprocessing.

## 1. Introduction

Trans-arterial radioembolization (TARE) with yttrium-90 (Y90) microspheres has been widely adopted as a primary locoregional treatment for patients with hepatocellular carcinoma (HCC) and liver-dominant hepatic metastases [1,2,3]. Evaluation of lung shunting fraction (LSF) is a critical step in Y90 treatment planning because increased intra-hepatic arterio-venous shunting poses a risk of lung damage and treatment failure [4]. Instead of lodging into the tumor microvasculature, the Y90 microspheres may travel through these shunts and into the lungs leading to radiation pneumonitis, especially if the absorbed radiation dose to the lungs exceeds 30 Gy in a single session or 50 Gy cumulative [5]. Moreover, lung shunting may decrease the Y90 radiation dose to the hepatic tumor and reduce the treatment effect [6,7,8]. Currently, LSF estimation requires a pre-treatment invasive arteriogram using Technetium-99m macroaggregated albumin (Tc-99m MAA) injected into the hepatic artery, followed by single-photon computed emission tomography (SPECT) imaging to determine the amount of Tc-99m MAA shunted to the lungs [9,10]. This LSF estimation using Tc-99m MAA SPECT assumes similar biodistribution in human body for Tc-99m-MAA and Y90 following hepatic arterial injection [11], and requires a dedicated hepatic artery catheterization in a separate earlier hospital visit increasing the costs in time, financial expense, and risk to patients [12]. Based on a recent analysis, the cost to Medicare of a visit for mapping angiography and SPECT imaging is $14,194 in 2021 US dollars [13]. A noninvasive, cost-effective method to estimate LSF without catheterization would improve the value of Y90 therapy to patients.

We hypothesize that LSF can be estimated from pre-TARE non-invasive imaging that sensitizes hepatic tissue blood flow. Tumors with higher arterio-venous shunting are expected to have higher blood velocities, as arterio-venous shunting bypasses small, high resistance vessels such as capillaries. Arterio-venous shunting requires tumor angiogenesis and may also change the plasma space and extravascular extracellular space (EES) volume. These changes can be captured by MR perfusion-weighted imaging such as dynamic contrast enhanced (DCE) MRI. Therefore, it may be possible to predict LSF by perfusion quantification from DCE MRI.

Recently, the quantitative transport mapping (QTM) method [14,15] has been developed to estimate mass flux characterized by velocity |u| without a global arterial input function (AIF) used in traditional Kety’s tracer kinetic analysis [16,17]. QTM velocity has been shown to be more accurate than Kety’s parameters in validation with numerical ground truth [14] and more sensitive than Kety’s parameters for differentiating benign from malignant tumors compared with biopsy [18,19]. Accordingly, we propose to investigate the feasibility of noninvasive prediction of LSF according to QTM velocity |u|, as well as Kety’s parameters, derived from DCE MRI.

## 2. Materials and Methods

### 2.1. Patients and MRI Protocols

This retrospective study was Institutional Review Board approved and HIPAA compliant. Twenty-five patients (age range: 42 to 87 years; 8 female and 17 male) were diagnosed with HCC as determined by Liver Imaging Reporting and Data System (LIRADS) [20]. All patients underwent trans-arterial radioembolization therapy utilizing Y90 resin or glass microspheres after diagnosis with the dose determined by either the body surface area (BSA) or medical internal radiation dose (MIRD) method. LSF was calculated from a single-photon computed emission tomography (SPECT) scan after Tc-99m-macroaggregated albumin (MAA) administration in the target hepatic artery [21,22]. Before Y90 treatment, the patients were scanned on a 1.5 T scanner (MAGNETOM, Siemens Medical Solutions, Erlangen, Germany) with 3D CAIPI-VIBE sequence before, during and after gadolinium contrast agent (Gd) injection [23]. Gd was injected with a dose of 0.1 mmol/kg (gadobutrol; Bayer Healthcare Pharmaceuticals Inc., Whippany, NJ, USA) at 2.0 mL/s, followed by a 20 mL saline flush using an MR compatible injector. The scanning parameters were as follows: In plane resolution 0.84 mm, slice thickness 3 mm, temporal resolution 5 s, repetition time 4.68 ms, echo time 2.39 ms, flip angle 10°.

### 2.2. Quantitative Perfusion Processing of DCE MRI

We assumed a linear relationship between tracer concentration and relative enhancement on DCE MRI [24]. The tracer concentration was then processed using quantitative transport mapping (QTM) and traditional kinetic modeling method. In QTM, the tracer concentration profile is modeled by a transport equation [14,25]:

(1) $$\partial_{t}c\left(r,t\right)=-\nabla \cdot c\left(r,t\right)u\left(r\right),$$

where c(r,t) is the tracer concentration scalar field, $r=\left(r_{x},r_{y},r_{z}\right)$ the spatial location in a volume of size $\left(N_{x},\;N_{y},N_{z}\right)$, $\partial_{t}$ the time derivative, $\nabla =\left(\partial_{x},\partial_{y},\partial_{z}\right)$ the spatial gradient operator, and time index $t\in \left\{1,2,\ldots N_{t}-1\right\}\;$the time index with $N_{t}$ as the number of time frames. $u\left(r\right)=\left(u^{x}\left(r\right),\;u^{y}\left(r\right),\;u^{z}\left(r\right)\right)$ is an average velocity vector field [25]. Both time derivative and gradient operator are implemented as finite differences in a discretized 4D spacetime-resolved image space. Equation (1) is a linear equation system for velocity that is solved as an optimization problem with L1 total variation regularization as in a recent QTM study with the regularization parameters $\lambda =10^{-4}$ chosen according to the L-curve method [14]:

(2) $$u=\underset{u}{\mathrm{argmin}}\sum_{t=1}^{N_{t}-1}\partial_{t}c+\nabla \cdot cu_{2}^{2}+\lambda \nabla u_{1}.$$

In traditional Kety’s tracer kinetics (also known as extended Tofts’ model), the tracer concentration profile is modeled by [26]:

(3) $$\partial_{t}c\left(r,t\right)=K^{\mathrm{trans}}\left(r\right)\left[c_{a}\left(t-\tau \right)-\frac{1}{V_{e}\left(r\right)}c\left(r,t\right)\right],$$

where $c_{a}\left(t\right)$ is the global AIF, $K^{\mathrm{trans}}\;$is volume transfer constant, $V_{e}\left(r\right)$ is the volume fraction of extravascular extracellular space (EES), and τ is traveling delay. Eqation (3) is a linear equation system for $K^{\mathrm{trans}}$ and $k_{ep}=\frac{K^{\mathrm{trans}}}{V_{e}}$, but is nonlinear to τ. A voxel-wise non-linear least squares method is used to solve for kinetic parameters and traveling delay τ of AIF with the regularization parameters λ = μ = $10^{-3}$ chosen according to the L-curve method [27,28,29]:

(4) $$K^{\mathrm{trans}},k_{ep},\tau =\underset{K^{\mathrm{trans}},k_{ep},\tau }{\mathrm{argmin}}\sum_{t=1}^{N_{t}-1}\|\partial_{t}c-K^{\mathrm{trans}}c_{a}\left(t-\tau \right)+k_{ep}c{\|}_{2}^{2}+\;\lambda \|\nabla K^{\mathrm{trans}}{\|}_{1}+\mu \|\nabla k_{ep}{\|}_{1}.$$

For each case, an experienced radiologist drew the AIF in the tumor-feeding artery and manually segmented regions of interest (ROI) consisting of the tumor targeted by Y90 radioembolization. |u|, $K^{\mathrm{trans}}$, $V_{e}$ and τ were averaged over these ROIs and used for further statistical analysis. All reconstructions were performed on a computer using an Intel i7-8700K 6-core CPU with 64GB memory.

### 2.3. Data Analysis

The patients were separated into 2 groups according to SPECT measured LSF values: a low-risk group (LSF ≤10%) and a high-risk group (LSF>10%) [30,31]. Tumors were manually segmented on post-Gd MRI to define lesion region of interest (ROI) and to measure tumor volume. Only the lesions that went through Y90 treatment were included in further study, and the average tumor size was 18.6 ± 15.7 cm^3^. Statistical analysis was performed on 6 parameters: ROI averaged |u|, $K^{\mathrm{trans}}$, $V_{e}$, τ, as well as tumor volume and patient age. A Spearman correlation test was performed to test the relationship between these parameters and lung shunt fraction. The Mann–Whitney U test was performed comparing these values between the low-risk and the high-risk groups. *p*-values at or below 0.05 were considered to indicate statistical significance. A receiver operating characteristic curve (ROC) analysis was performed to investigate the risk prediction performance of all parameters, and the optimal threshold was calculated by maximizing sensitivity plus specificity. Statistical analysis was performed using the R Statistical Software (Foundation for Statistical Computing, Vienna, Austria).

## 3. Results

All 25 patients successfully underwent DCE MRI, SPECT and Y90 treatment. QTM |u| and Kety’s $K^{\mathrm{trans}},\;V_{e},\tau$ maps were successfully reconstructed from DCE MRI images. Twelve subjects with LSF ≤10% were labeled as low LSF group and thirteen subjects with LSF>10% were labeled as high LSF group. Between the low LSF and high LSF group, there was no significant difference in patient age (68.16 ± 11.87 vs. 65.30 ± 12.47, *p* = 0.4791) and tumor volume (0.16 ± 0.08 vs. 0.32 ± 0.18 mm/s, *p* = 0.7237).

**Figure 1:**
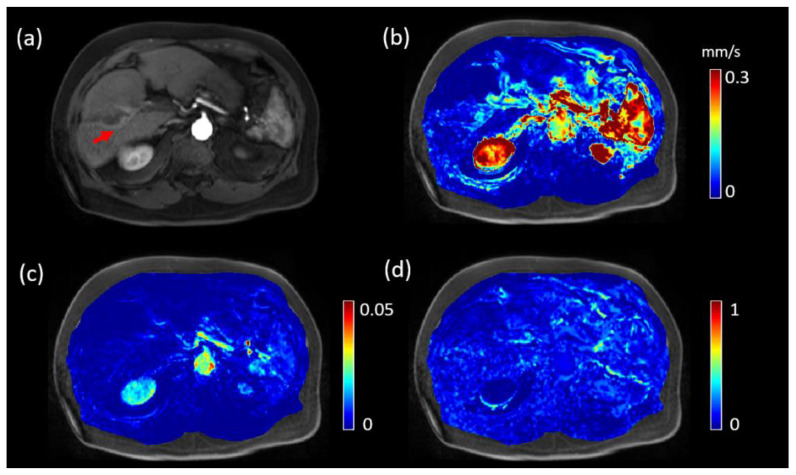
DCE MRI, QTM velocity, $K^{trans}$ and $V_{e}$ maps of HCC of a 74-year-old patient with lung shunting fraction 9.3%. (**a**) post-Gd T1 weighted image showing the tumor (red arrow). (**b**) QTM |***u***| map, (**c**) $K^{trans}$ map, and (**d**) $V_{e}$ map.

**Figure 2:**
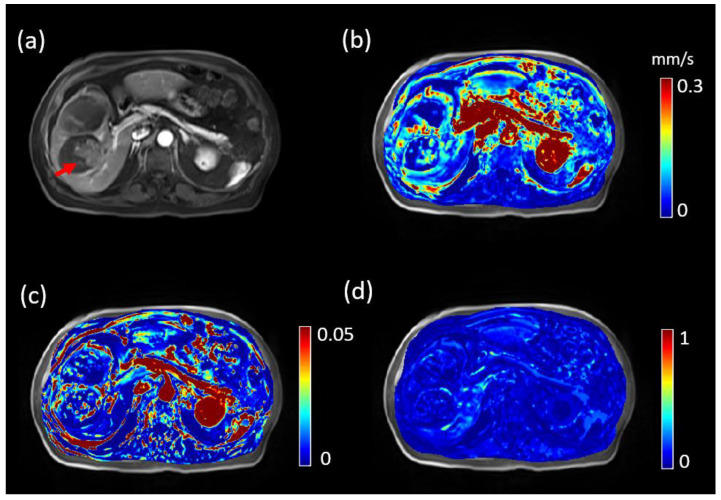
DCE MRI, QTM velocity, $K^{trans}$ and $V_{e}$ maps of HCC of a 78-year-old patient with lung shunting fraction 15.3%. (**a**) post-Gd T1 weighted image showing the tumor (red arrow). (**b**) QTM |***u***| and $V_{e}$ map, (**c**) $K^{trans}$ map, and (**d**) $V_{e}$ map.

Figure 1 shows a representative low LSF case of a 74-year-old patient (LSF = 9.3%, tumor volume 31.03 cm^3^). The lesion demonstrated enhancement on post-contrast DCE MRI (red arrow in Figure 1a), and processed |u|, $K^{\mathrm{trans}}$ and $V_{e}$ maps are shown in Figure 1b–d, respectively. For QTM, |u| was 0.06 mm/s, while for Kety’s method $K^{\mathrm{trans}\;}$= 0.0033, $V_{e}$ = 0.1073 and τ=15.86 s. As a comparison, Figure 2 shows similarly a representative high LSF case of a 78-year-old patient (LSF = 15.3%, tumor volume 14.43 cm^3^). For QTM, |u| = 0.09 mm/s, while for Kety’s method $K^{\mathrm{trans}}\;$= 0.0313, $V_{e}$ = 0.0560 and τ=15.82 s.

**Figure 3:**
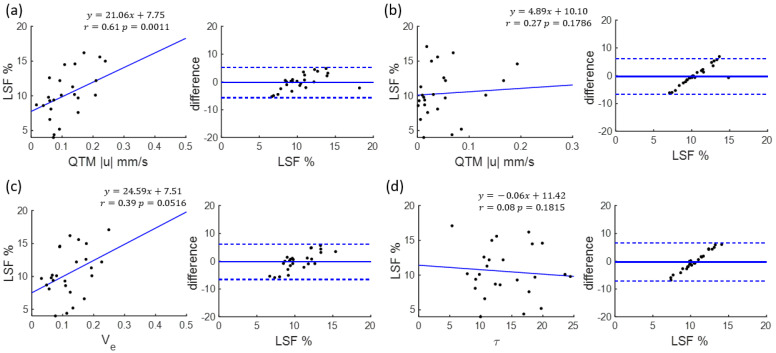
Spearman correlation and Bland–Altman plot of (**a**) QTM |u|, (**b**) $K^{trans}$ (**c**) $V_{e}$, (**d**) τ with lung shunting fraction (LSF).

Table 1 summarizes the correlations of LSF with QTM |u| and Kety’s $K^{\mathrm{trans}},\;V_{e},\tau$, as well as tumor volume and patient age. Only QTM |u| demonstrated significant correlation with LSF (r = 0.6156, F = 14.0363, *p* = 0.0011). No significant relationship was found for $K^{\mathrm{trans}}$(r = 0.2778, F = 1.9251, *p* = 0.1786), $V_{e}$(r = 0.3936, F = 4.2168, *p* = 0.0506), τ(r = 0.0883, F = 0.1815, *p* = 0.6741), tumor volume (r = 0.2735, F = 0.1858, *p* = 1.8603), or age (r = 0.2272, F = 0.2749, *p* = 1.2508). The details of linear regressions and corresponding Bland–Aleman plot are illustrated in Figure 3 and Table 1.

**Figure 4:**
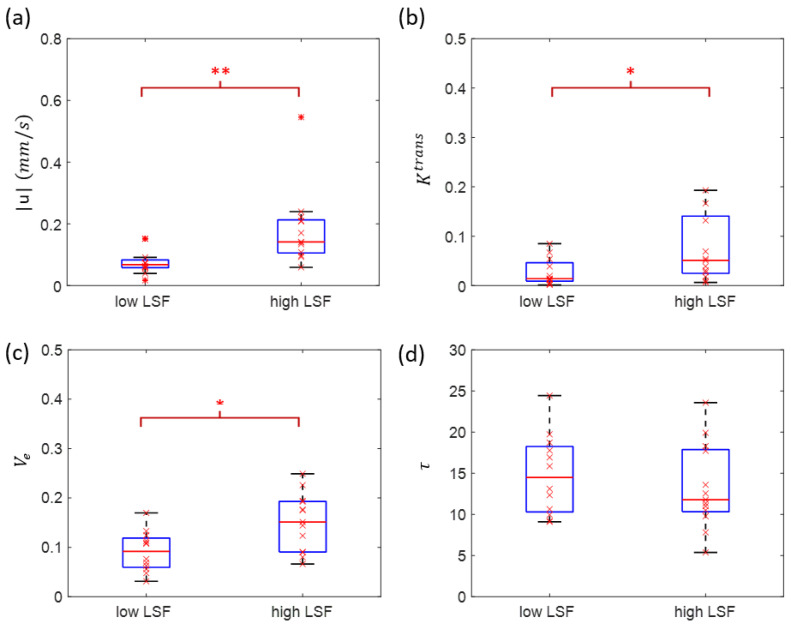
Mann–Whitney U test of QTM |u| (**a**), $K^{trans}$ (**b**), $V_{e}$ (**c**), τ (**d**) between low-risk subjects (lung shunting fraction ≤10%) and high-risk subjects (lung shunting fraction >10%). (LSF: lung shunting fraction; *: *p* < 0.05; **: *p* < 0.01).

**Figure 5:**
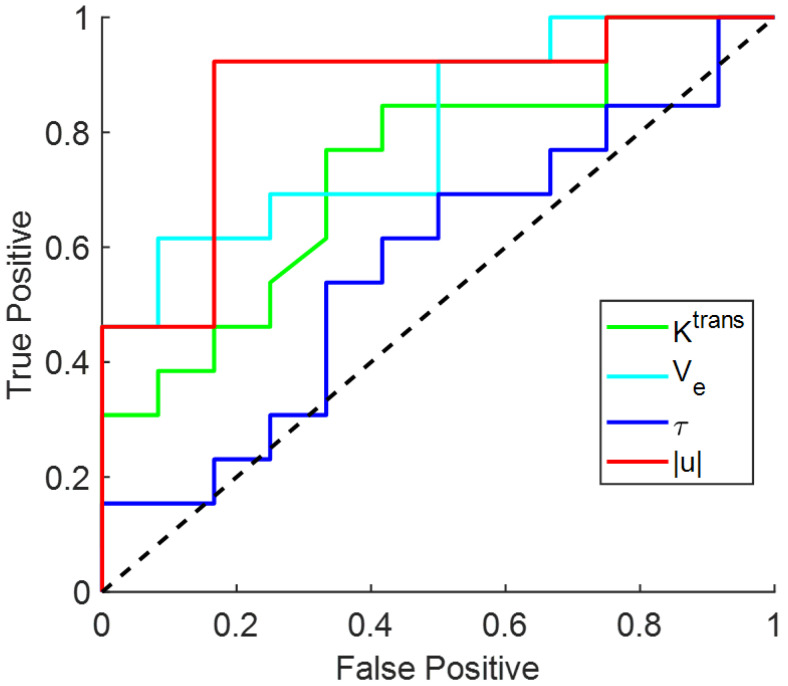
Receiver operating characteristic curve (ROC) for QTM |u|, $K^{trans},V_{e},\;\tau$ in differentiating low-risk (lung shunting fraction ≤10%) and high-risk (lung shunting fraction >10%) subjects.

Figure 4 illustrates the U test results. There was a significant difference between low LSF group and high LSF group for |u| (0.0760 ± 0.0440 vs. 0.1822 ± 0.1225 mm/s, *p* = 0.0011),$\;K^{\mathrm{trans}}$ (0.07 ± 0.04 vs. 0.07 ± 0.06, *p* = 0.04) and $V_{e}$ (0.0900 ± 0.0307 vs. 0.1495 ± 0.0485, *p* = 0.0114). No significant difference was found for τ (14.43 ± 3.86 vs. 13.12 ± 3.87 s, *p* = 0.5679). Figure 5 illustrates corresponding ROC analysis. The AUC for predicting low versus high risk was highest for |u| (AUC = 0.87, 95% confidence level 0.63–0.97), followed by $V_{e}$ (AUC = 0.80, 95% confidence level 0.56–0.93), $K^{\mathrm{trans}}$ (AUC = 0.74, 95% confidence level 0.49–0.90), τ (AUC = 0.57, 95% confidence level 0.32–0.82), tumor volume (AUC = 0.54, 95% confidence level 0.30–0.76) and patient age (AUC = 0.59, 95% confidence level 0.32–0.80), which are summarized in Table 2 along with sensitivity and specificity. The AUC of QTM |u| is significantly higher than AUC of patient age (*p* = 0.02), τ (*p* = 0.01) and tumor volume (*p* = 0.01), but not significantly higher than AUC of $V_{e}$ (*p* = 0.27) and $K^{\mathrm{trans}}$ (*p* = 0.15).

## 4. Discussion

These preliminary data from 25 patients undergoing Y90 radioembolization of HCC demonstrate a significant correlation between QTM velocity |u| derived from DCE MRI and lung shunt fraction (LSF) derived from SPECT post-Tc-99m-MAA administration, suggesting the potential for using noninvasive DCE MRI to predict LSF for distinguishing low lung shunting risk subjects (LSF ≤ 10%) from high lung shunting risk subjects (LSF > 10%). This result encourages the development of noninvasive imaging, including DCE MRI with QTM velocity |u| postprocessing, for accurate estimation of LSF to eliminate the cost and risk of catheterization in planning trans-arterial radioembolization with Y90 treatment.

Lung shunting is caused by the vasculature remodeling with tumor progression, creating high flow arterio-venous shunts that bypass high resistance capillaries [32,33]. The changes in tumoral vascularity may increase blood flow, velocity, plasma space and EES space volume, which are reflected on DCE MRI images and quantified by transport physics. In this study, we compared QTM |***u***| with parameters from traditional kinetic modeling (also known as extended Toft’s model) [26]. In the patient cohort, QTM velocity |u| but not Kety’s parameters demonstrated significant correlation with LSF. Although Kety’s method has not been applied to lung shunting fraction estimation in previous studies, this result is consistent with the hypothesis that increased artery–vein connections bypassing capillaries (shunts) increases the mean liver blood velocity, and also consistent with previous reports showing QTM improves upon Kety’s method by replacing a global arterial input function in Kety’s model with the local mass flux gradient in QTM [14,18,19]. In addition to a significantly higher velocity |u| observed in high LSF group, we also observed an increase in Kety’s parameters $K^{trans}$ and $V_{e}$ in high LSF group, which may reflect a higher tissue exchange rate and EES space in tumors with abnormal vasculature compared to normal tissue [34,35]. These observations should be evaluated in further studies, especially using histopathology for validation.

The LSF prediction accuracy may be further improved by increasing the spacetime resolution of DCE MRI [36]. Other MRI sequences that reflect vasculature properties such as arterial spin labeling, 3D/4D phase contrast MRA and susceptibility weighted imaging may also be incorporated into noninvasive LSF prediction. The artery–vein shunting process may change the arterial and venous blood volume and the average vessel size in a voxel. Velocity-sensitive pulses such as multiphase balanced steady-state free precession (bSSFP) pulse train and velocity-selective RF pulse train can be used to directly measure arterial and venous blood volume [37,38,39]. The average vessel size in the voxel can be estimated by measuring the R2 and R2* changes after intravascular superparamagnetic contrast agent injection [40,41,42]. These sequences may allow detailed microvasculature modeling and computational fluid dynamics prediction of the passage of Y90 microspheres through arteriovenous shunting [14].

Accurate LSF prediction may require detailed image features and microcirculation information in addition to simple lesion ROI values used in this study. For example, texture analysis or radiomic features of the transport quantity maps, deep learning and larger data sets may help construct the LSF prediction model. We combined QTM velocity ROI values with Kety’s parameter ROI values but the combination failed to improve the LSF prediction accuracy, which may suggest that QTM already contains all the information in Kety’s parameters as well as better information than Kety’s parameters, consistent with previous publications [14,18,19]. Historically, Tc-99m-MAA has been a well-developed radiotracer for pulmonary vascular imaging, especially for studying pulmonary embolism since 1960s [43] and has been conveniently adopted for estimating LSF since the beginning of radioembolization practice [44,45,46]. Fundamentally, the use of LSF assumes the good agreement between pretreatment Tc-99m-MAA distribution and final Y90-microsphere distribution. However, this assumption is highly problematic [47] since there may be differences in catheter position between the two hospital visits, physiologic variances in hepatic blood flow, and size and morphology differences between Tc-99m-MAA particles and Y90-microspheres. Accordingly, future development should focus on predicting biodistribution of Y90 microspheres based on maps of transport quantities such as using computational fluid dynamics (CFD) modeling [48,49]. This CFD modeling would estimate both Y90 microsphere distribution in the targeted liver region and Y90 microsphere scape into the lung.

There are several limitations in this study that need to be addressed in the future work. Firstly, the sample size is relatively small (*n* = 25), thus no significantly difference in AUC between QTM parameters and Kety’s parameters was observed. More cases will more thoroughly evaluate the LSF prediction performance of our proposed model. Secondly, our DCE MRI images contain only five temporal points and cover only 5 s of the arterial phase after Gd injection. A longer scan with higher spacetime resolution acquired with motion compensation [50,51] may help to increase the reconstruction accuracy of quantitative transport quantities. Thirdly, the assumption of linear relationship between tracer concentration and relative enhancement of DCE MRI may be problematic, which can be improved using quantitative susceptibility mapping [52,53].

In conclusion, noninvasive LSF prediction from DCE MRI with quantitative transport mapping postprocessing is feasible. Future work should include enlarging the dataset and exploring the combination of DCE MRI and other MR pulse sequences to improve the LSF model performance for predicting Y90 biodistribution.

## Figures and Tables

**Table 1:** Spearman correlation with LSF.

	R (95% CI)	*p* Value	F Value
QTM |***u***|	0.6156 (0.4148–0.8164)	**0.0011**	14.0363
$K^{\mathrm{trans}}$	0.2778 (0.0244–0.5312)	0.1786	1.9251
$V_{e}$	0.3936 (0.1403–0.6469)	0.0516	4.2168
τ	0.0883 (−0.0921–0.2687)	0.1815	0.6741
Tumor volume	0.2735 (0.0206–0.5264)	0.1858	1.8603
Age	0.2272 (−0.0180–0.4724)	0.2749	1.2508

**Table 2:** ROC analysis for low-risk and high-risk LSF classification.

	AUC (95% CI)	Sensitivity (95% CI)	Specificity (95% CI)
QTM |***u***|	0.87 (0.63–0.97)	0.92 (0.63–1)	0.83 (0.5–1)
$K^{\mathrm{trans}}$	0.74 (0.49–0.90)	0.77 (0.42–0.93)	0.67 (0.30–0.89)
$V_{e}$	0.80 (0.56–0.93)	0.62 (0.30–0.85)	0.92 (0.54–1)
τ	0.57 (0.32–0.82)	0.54 (0.23–0.80)	0.58 (0.29–0.88)
Tumor volume	0.54 (0.30–0.76)	0.31 (0.08–0.66)	0.92 (0.63–1)
Age	0.59 (0.32–0.80)	0.62 (0.31–0.85)	0.67 (0.32–0.9)

## Data Availability

The data presented in this study are available on request from the corresponding author.
